# Associated factors of diet quality among people living with HIV/AIDS in Ghana

**DOI:** 10.1186/s40795-024-00898-y

**Published:** 2024-06-21

**Authors:** Kasim Abdulai, Kwasi Torpey, Agnes Millicent Kotoh, Amos Laar

**Affiliations:** 1 Translational Nutrition Research Group, Department of Clinical Nutrition and Dietetics, University of Cape Coast, Cape Coast, Ghana; 2 Department of Population, Family and Reproductive Health, School of Public Health, University of Ghana, Legon, Accra Ghana

**Keywords:** HIV, Diet quality, Individual dietary diversity score, Associated factors

## Abstract

**Introduction:**

: Nutrition is a very important element of a comprehensive care for people living with HIV/AIDS (PLHIV), especially in resource-constrained settings where malnutrition and food insecurity are common. Dietary diversity is a useful indication of nutritional adequacy (diet quality) in people of all ages. An optimally diverse diet strengthens the body’s immune system.

**Objective:**

This study aimed to assess diet quality and its associated factors among PLHIV.

**Methods:**

A facility-based cross-sectional study design was employed to select 440 PLHIV from two hospitals in the Eastern Region of Ghana. Dietary intakes were determined using 24-hour recall. A stadiometer and bioimpedance analysis machine were used to obtain anthropometric and body composition data. Diet quality was assessed using FAO’s individual dietary diversity score (IDDS) as a proxy. *SPSS* version 20 was used for analysis. Odds ratios and ordinal logistic regression were used to identify factors associated with diet quality among the PLHIV. P-value was set at 0.05.

**Results:**

Most of the PLHIV (73%) consumed from ‘Starchy staple” food group. Less than 20% of the study sample consumed ‘Fruits’ and ‘Vegetables’ (17% and 14% respectively) a day before the survey. The mean IDDS was 4.11 (SD = 1.29). Overall, most of the PLHIV (56%) had medium IDDS which is equivalent to “diet needing improvement’, 14% had higher IDDS (good diet), whiles about 31% of the participants actually had poor diet (lower IDDS). Associated factors of diet quality were age (AOR = 0.966: 95%CI: 0.936–0.997: p = 0.031), married (AOR = 4.634: 95%CI: 1.329–16.157: p = 0.0016), separated (AOR = 0.0203: 95%CI: .036–0.994: p = 0.049), and daily meal frequency (AOR = 0.441: 95%CI: .478–1.948: p = 0.020). Overall, the model accounts for about 20% of the variation in diet quality of the participants (pseudo-R square = 0.196).

**Conclusion:**

This study demonstrates that most of the PLHIV did not consume good diet which may have an implication on their immune system, which is already under attack by HIV, and probably emerging infections. Age, marital status, and meal frequency were the variables that predicted diet quality among the study participants.

## Introduction

Malnutrition and HIV/AIDS are both quite common in many regions of the world, particularly in Sub-Saharan Africa [1]. These are sometimes referred to as a syndemic as the consequences are interconnected and reinforce each other [2]. HIV has a direct impact on nutrition status by escalating energy demands, lowering food intake, and impairing nutrient uptake and utilization [3].

Nutrition is a critical component of comprehensive care for people living with HIV/AIDS (PLHIV), especially in resource-constrained settings where malnutrition and food insecurity are common [4].

The immune system is impaired by a decrease in CD4 T cells, suppression of delayed hypersensitivity, and altered B-cell responses, which are comparable to the cellular effects of starvation and HIV [5]. Delivering enough food and nutrition to meet people’s fundamental needs for wellness, growth, and development has long been a difficulty in Africa [4, 6, 7],

Also, some of the earlier symptoms of HIV infection that were observed included nutritional deficiencies [8]. These issues arise as a result of insufficient dietary intake and altered metabolic circumstances, resulting in an imbalance of energy and nutrients in patients who are receiving antiretroviral medication (ART) [9]. This poor nutritional situation has a greater impact on PLHIV because they are more susceptible to opportunistic illnesses [10].

Dietary management of PLHIV is therefore critical to maintaining their ability to continue working and contributing to socioeconomic development of the country. Again, food insecurity and malnutrition can hasten the onset of AIDS-related diseases [11]. Diet plays a significant role in the immune system of HIV/AIDS patients, because people living with HIV require sufficient amounts of macro- and micronutrients to function normally [12, 13]. Thus, there is the need to improve food security among the people living with HIV.

Food security exists when “all people, at all times, have physical and economic access to sufficient, safe and nutritious food to meet their dietary needs and food preferences for an active and healthy life” [14]. Diet quality refers to a diversified, balanced, and healthy diet that provides energy and all essential nutrients for growth and a healthy and active life [15]. It is a key component of the definition of food security, and measuring diet quality is of growing relevance [16]. In this study, individual dietary diversity score (IDDS) was used as a proxy to measure diet quality [17, 18]. A varied diet is necessary for achieving key dietary requirements, especially for people who are at risk of nutrient deficiencies that can lead to malnutrition [19].Dietary diversity scores are based on a basic count of food groups consumed by a household or an individual in the previous 24 h [20]. Various anthropometric measures and nutritional intakes have been linked to dietary diversity score [21].

Ghana is a lower-middle income country (LMIC) undergoing rapid dietary/nutrition transition. This transition reflects substantial changes in the country’s food consumption patterns, dietary habits, and nutritional status, driven by various socio-economic factors and lifestyle shifts. As Ghana experiences economic growth and urbanization, there is a noticeable transformation in the traditional food landscape, characterized by increased access to a variety of foods, changes in dietary preferences, and alterations in eating habits. This nutritional transition is often associated with a rise in the prevalence of non-communicable diseases and lifestyle-related health issues. These changes are taking place across both rural and urban regions [19] This is a concern to public health, with a disproportionately negative impact on the wellbeing and nutritional status of the poor and other people with increased nutritional risk such as PLHIV.

As a result, assessing the overall diet quality is critical. Dietary variety has grown in popularity as a tool for assessing food diversity, owing to its validity, ease of measurement, and low cost of implementation in developing countries [19]. Dietary diversity is also a useful indication of nutritional adequacy (diet quality) in people of all ages, according to researchers [4, 22, 23].

This study assessed the diet quality of PLHIV using IDDS, and also explored factors that predicted diet quality among the PLHIV.

## Methods

### Study design and settings

A facility based cross-sectional study design was adopted. The research was carried out between February 2020 and June 2020. People living with HIV (PLHIV) who were 18 years or older and had been on antiretroviral therapy (ART) for at least 6 months were included in the study. St. Martins de Porres Hospital in Agomanya and Atua Government Hospital in Atua which provide ART services, both facilities are located in the Lower Manya Krobo District of the eastern region, Ghana, were the study sites. PLHIV who met the study’s inclusion criteria including being able to give informed written consent and agreed to participate in the study were chosen.

The exclusion criteria for selecting the PLHIV were: PLHIV who were pregnant and lactating; PLHIV on special diet; and PLHIV who visit either of the two hospitals but not for ART.

### Study variables

The study’s main outcome variable was individual dietary diversity score (diet quality). Explanatory variables of the study included alcohol consumption, ART drug type, duration of exposure to ART, smoking, age, sex, gender, level of education, and exercise.

### Sample size determination

The required sample size was calculated using a formula for determining sample size for a single population proportion [20], using 50% [21] as the proportion of low dietary diversity (P) with a 5% level of significance, at a 95% level of confidence for a two-tail test, and a marginal error or level of precision (d) = 5%. The sample size (n) was determined as follows:

$$\begin{gathered}n = \frac{{{Z^2}*P(1 - P)}}{{{d^2}}} \hfill \\\,\, = \frac{{{{1.96}^2}*0.5(1 - 0.5)}}{{{{0.05}^2}}} = 384 \hfill \\ \end{gathered}$$

The minimum sample size of 384 was therefore sufficient to answer the research question. However, 15% was added in order to adjust for nonresponse rate and missing data resulting in a final sample of **440**.

### Sampling procedure

The sampling procedure for this study involved selecting a total of 440 participants from two hospitals, St. Martins De Porres Hospital and Atua Government Hospital, using a method that ensures a representative sample of the patient populations at these facilities. The method employed was Probability Proportional to Size (PPS), which allocates the sample according to the volume of active patients at each hospital. This allocation was determined by calculating the proportion of active patients at each hospital relative to the total number of active patients at both hospitals. The resulting fractions were then applied to the total sample size, allocating 228 participants to Atua Government Hospital and 212 to St. Martins De Porres Hospital.

A random sampling technique was used during Antiretroviral Therapy (ART) clinic days. Observations indicated that about 50 patients living with HIV (PLHIV) attended Atua and about 60 attended St. Martin De Porres on clinic days. To meet the sample allocation, 20 participants were randomly selected at each ART clinic day at Atua and 25 at St. Martin De Porres until the totals of 228 and 212 were respectively achieved. The random selection process involved patients drawing from a box containing slips marked “YES” or “NO.” Atua’s box contained 20 “YES” slips, and St. Martin De Porres’s box contained 25 “YES” slips, corresponding to the number of participants needed per clinic day. Those who drew a “YES” slip and provided consent were included in the study.

This sampling strategy was meticulously designed to reflect the diversity and characteristics of the hospital patient populations, ensuring that the study findings could be generalized to the broader patient community served by these institutions. The use of PPS for sample distribution accounted for the relative patient volumes at each hospital, and the random selection method minimized selection bias, enhancing the study’s validity.

### Data collection methods and procedures

Data on sociodemographic characteristics such as gender, age, ethnicity, religion and occupation were collected using a questionnaire. Dietary intake of participants was measured with a 24- hour recall of usual food intake. Detailed information on all meals, snacks, and beverages consumed in the past 24 h was obtained. It required subjects to remember the specific foods as well as quantities consumed in the past 24 h.

The 24-hour dietary recall method was selected for this study due to its practicality and efficiency in rapidly collecting detailed dietary information from a large sample. This method is advantageous for its cost-effectiveness and minimal burden on participants, requiring them only to recall their food intake for a single preceding day. Unlike continuous tracking methods such as food diaries, the 24-hour recall does not risk altering participants’ normal eating habits due to the awareness of being monitored. Additionally, it offers flexibility in data collection, adaptable to face-to-face and digital administration, which is particularly useful in diverse population settings.

Techniques such as strategic prompting to help the subject recall any drinks, snacks, condiments, etc. that may otherwise be forgotten were applied. Respondents were asked to report portion size based on standard sizes (e.g., one soup ladle of porridge) and/or using food models to improve accuracy of portion size estimation.

### Diet quality

As outlined earlier within this paper, individual dietary diversity scores (IDDS) was chosen as a proxy for diet quality [17, 18, 24]of the participants. The diet quality of the study participants was determined using data on their usual food intakes from the 24-Hour Recall. The IDDS was used as a proxy to determine the quality of their diet [27–36]. The IDDS used was a modified version of the Food and Agricultural Organization (FAO) dietary diversity questionnaire [24]. The FAO dietary diversity questionnaires is a 12-item scale designed to assess the variety of the diet by summing the number of food groups eaten by household members but uses 9-item scale for individuals in the last 24 h [24, 25]. The 12 major food groups inquired about are vegetables, fruits, cereals, meat, fish, tubers, legumes, eggs, milk and milk products, fats and oils, sugar and sweets, beverages. The reference period can either be the previous day or week [24].

At a household level, the dietary diversity score serves as an indicator of food accessibility, reflecting a household’s ability to obtain diverse and sometimes expensive food groups. On an individual level, individual dietary diversity scores (IDDS) offer straightforward and validated metrics for assessing dietary quality and nutrient sufficiency [24]. In this study, the IDDS of PLHIV was derived on the basis of the number of food groups consumed from a 24-hour recall. Any food group consumed in the past 24 h was given a score of one [1] which was aggregated to give the IDDS, with a maximum possible score of nine [9]. According to FAO categorization, a score of zero (0) was assigned to a food category if not consumed in the past 24 h. A score of 3 or less indicates lower IDDS (poor diet), a score of 4 and 5 indicates medium IDDS (diet needing improvement), and a score of 6 or more indicates high IDDS (good diet) [24].

### Quality control

Prior to the study, Research Assistants were trained and tested for competence in using study instruments and tools. Research equipment underwent validation by calibrating and testing known measures for validity and reliability. To safeguard participant confidentiality, non-local research assistants were employed. They attended a two-day workshop led by the principal investigator. The workshop covered study objectives, sampling techniques, data collection tools usage, and ethical considerations. Confidentiality, HIV sensitivity, and safety protocols were emphasized. Outcome and explanatory variables were pre-defined (coded, labeled and assigned values) in the data entry template. The collected data were then transferred into MS excel 2016 and saved in a file on a laptop only accessible to the researcher.

### Data management and analysis

Mobile phones with the help of *ODK Collect App* were used to collect the data. Data collectors reviewed and corrected errors in completed data entries before submission, following a data cleaning protocol. The principal investigator (PI) then verified accuracy, consistency, and completion. The collected data was exclusively accessible to the PI. For consistency and validation, data were exported to IBM SPSS Statistics 20 [26, 27],. The same program was used to analyze cleaned and validated datasets.

Utilizing univariate analysis, we created descriptive tabulations for key variables. Associated factors of the primary outcome (diet quality) were identified through an ordinal multivariable logistic regression model, incorporating socio-demographic and clinical attributes of PLHIV as potential associated factors.

Variables that exhibited a *p* < 0.20 in the bivariate analysis were selected for inclusion in the multiple regression model. Several factors, as detailed in Table 1, were considered as potential associated factors. Pre-identified confounding variables such as Sex, occupation, and exercise status were included. We utilized the ordinal logistic regression modeling technique with the “Enter” method in SPSS following a stepwise process. All variables were entered simultaneously into a single full model generated in a single step. A significance level of *P* < 0.05 was considered to indicate statistical significance.

**Table 1:** Dietary diversity (diet quality) and anthropometric/body composition parameters

Variable	Estimate	95% CI	*p*-value
BMI	0.12	-0.03–0.28	0.13
Percentage body fat	0.05	-0.02 - -0.13	0.16
Percentage muscle mass	0.03	-0.08–0.15	0.59
Visceral fat	-0.18	-0.34 - -0.02	0.03
Weight	0.05	0.01–0.10	0.04

Table 1 presents the relationships between dietary diversity (diet quality) and various anthropometric and body composition parameters. The table includes estimates of the association, 95% confidence intervals (CIs), and p-values for each variable.

### Ethical considerations

This study received ethical approval from the Ghana Health Service Ethics Review Committee (GHS-ERC) under protocol number GHS-ERC 007/07/19. Participants provided informed consent by signing before engaging in the study. For those unable to read and write, consent documents were read and explained in a language they understood, and consent was recorded with a thumbprint, a method approved by the GHS-ERC. Throughout the research process, we ensured strict confidentiality and privacy of participant data. Additional measures were implemented to safeguard data security during and after the study. These measures included secure storage of digital data with encrypted access, and all physical forms were kept in locked, secure locations to prevent unauthorized access, thereby reinforcing the protection of participant information and adhering to ethical standards.

## Results

### Demographic characteristics of participants

Table 2 presents the socio-demographic characteristics of the study participants. A total of four hundred and forty (*n* = 440) PLHIV were selected. There were more females (85%) than males (15%). The mean age of the participants was 49.28 years (SD = ± 11.96). More than half of the participants (57%) belonged to the age group 35–54 years. About a quarter (25%) had never been to school and only about 3% had attained educational level higher than secondary.

One hundred and thirty-seven (31%) of the PLHIV who participated in the study were married, followed by those who were unmarried (29%). One hundred and twenty-seven (29%) were widowed and 11% were either divorced or separated.

The participants were also categorized based on their occupational status. In this respect, majority (58%) of them are into trading. Participants were predominantly Ga/Dangmes (81%), followed by Ewes (10%), Akans (6%), and about 3% belonged to other ethnic groups. Again, participants were predominantly Christians (98.2%). There were six (1.4%) Muslims and two (0.5%) Traditionalists (Table 2).

**Table 2:** Socio-demographic characteristics of participants

Characteristics	Female, *n* (%) *373 (85)	Male, *n* (%) **67 [15]	Total, *n* (%) 440 (100)
**Facility**			
Atua	197 (52.7)	32 (47.8)	229 (51.9)
St. Martins	177 (47.3)	35 (52.2)	212 (48.1)
**Age Group**			
18–34 years	43 (11.5)	7 (11.5)	50 (11.3)
35–54 years	225 (60.2)	25 (37.3)	250 (56.7)
55 + years	106 (28.3)	35 (52.2)	141 (32.0)
**Level of Education**			
None	108 (28.9)	3 (4.5)	111 (25.2)
Primary	73 (19.5)	12 (17.9)	85 (19.3)
Middle/Secondary	159 (42.5)	40 (59.7)	199 (45.1)
Higher	9 (2.4)	4 (6.0%)	13 (2.9)
**Marital Status**			
Single	114 (30.6)	14 (20.9)	128 (29.1)
Married/Cohabiting	100 (26.9)	37 (58.3)	137 (31.1)
Divorced/Separated	43 (11.6)	5 (7.5)	48 (10.9)
Widowed	116 (31.1)	11 (16.4)	127 (28.9)
**Occupation**			
Unemployed	44 (11.8)	5 (7.5)	49 (11.1)
Farmer	14 (3.7)	25 (37.3)	39 (8.8)
Artisan	41 (11.0)	24 (35.8)	65 (14.7)
Formal Public Sector	19 (5.1)	2 (3.0)	21 (4.8)
Formal Private Sector	5 (1.3)	4 (6.0)	9 (2.0)
Trading	250 (66.8)	5 (7.5)	255 (57.8)
Others	1 (0.3)	2 (3.0)	3 (0.7)
**Ethnicity**			
Akan	22 (5.9)	4 (6.0%)	26 (5.9)
Ga/Dangme	302 (80.7)	57 (85.1%)	359 (81.4)
Ewe	40 (10.7)	4 (6.0%)	44 (10.0)
Others	10 (2.7)	2 (3.0%)	12 (2.7)
**Religion**			
Christianity	367 (98.1)	66 (98.5%)	433 (98.2)
Islam	5 (1.3)	1 (1.5%)	6 (1.4)
Traditional	2 (0.5)	0 (0.0%)	2 (0.5)

This table presents a detailed breakdown of the socio-demographic characteristics of 440 participants who participated in the study. The mean age of the participants was 49.28 years (SD = ± 11.96). * = Total number of females (*n* = 373); ** = Total number of males (*n* = 67);

### Consumption of different food groups by the PLHIV

**Fig. 1:**
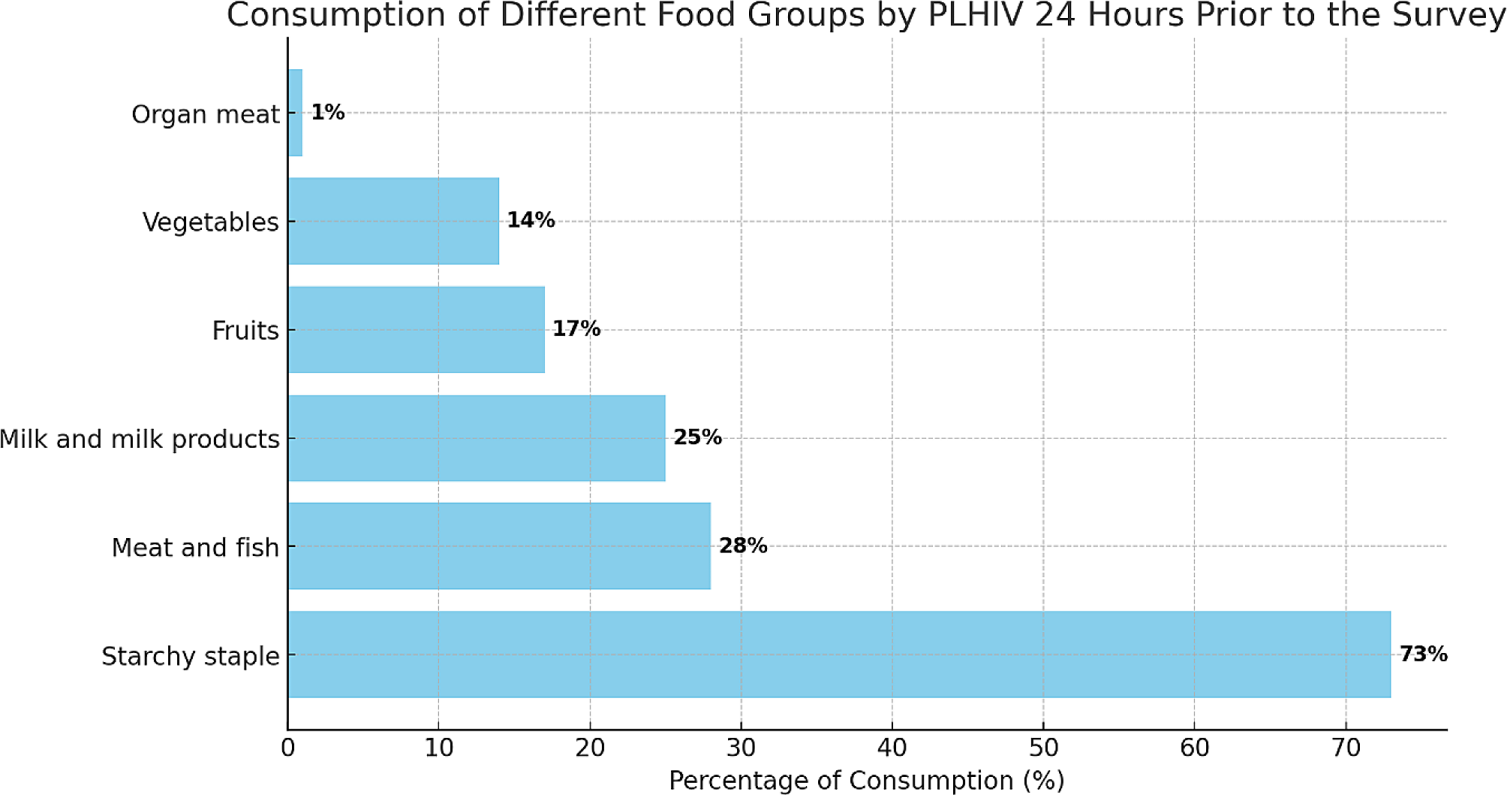
Consumption of different food groups 24 h prior to the survey. The chart presents the consumption patterns of different food groups by people living with HIV (PLHIV) in the 24 h preceding a survey

In Fig. 1, the depiction illustrates the consumption of foods from various food groups by the study population during the day prior to the survey. Taking into account the dietary diversity and consumption habits of the participants, majority of people living with HIV (73%) reported having consumed items from the “Starchy staple” food group within the 24 h leading up to the interview. About 28% of the participants had used ‘Meat and fish’ in the previous 24 h, whiles about 25% of the study population consumed ‘Milk and milk products. Overall, fruits and vegetables consumptions were not satisfactory. Less than 18% of the study sample had consumed ‘Fruits’, ‘Vegetables’, and ‘Organ meat’; 17%, 14%, and 1% respectively.

### Examining diet quality (IDDS) among study participants

**Fig. 2:**
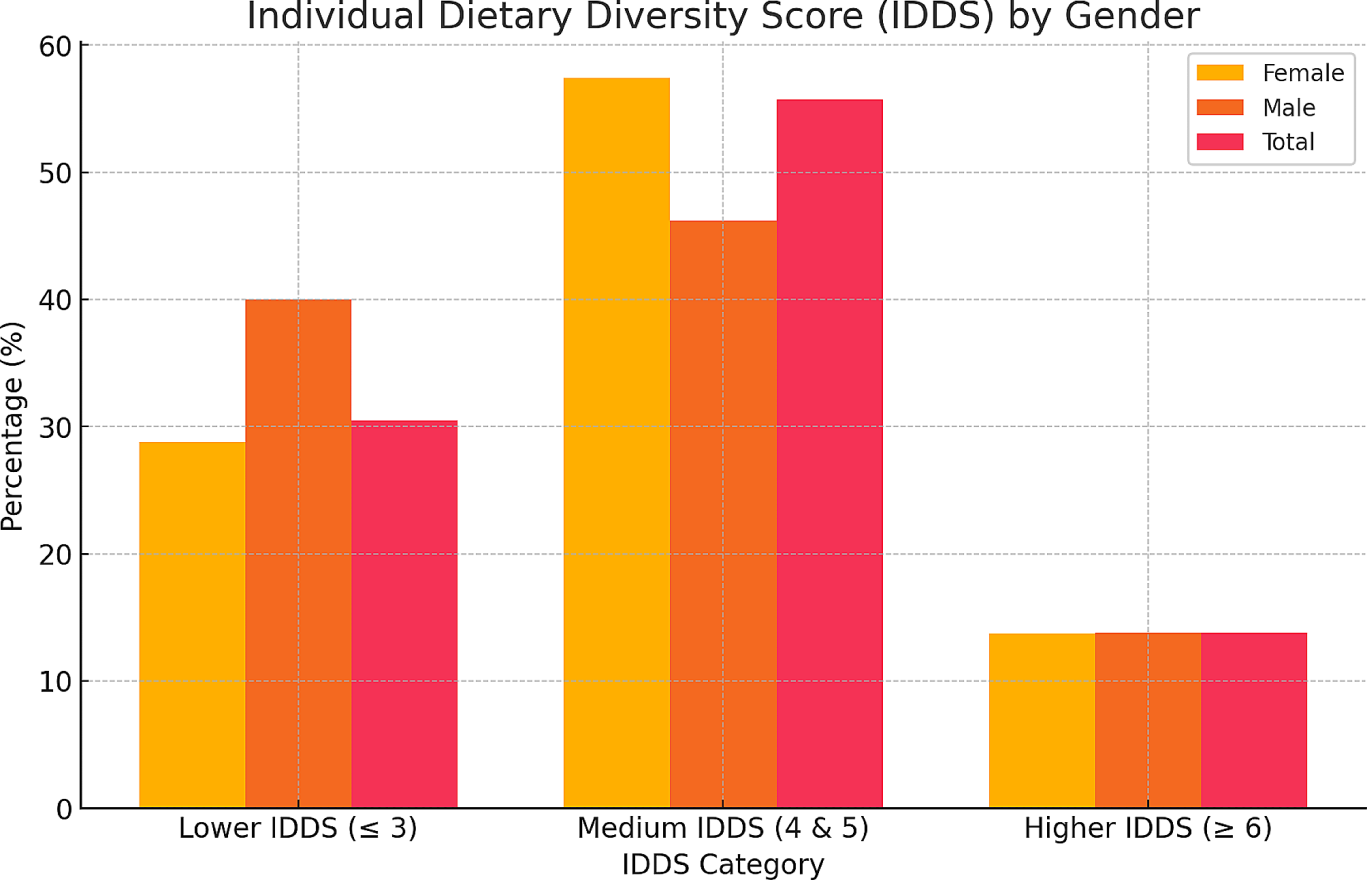
Individual Dietary Diversity Score (IDDS) of study participants. The bar chart illustrates the distribution of Individual Dietary Diversity Scores (IDDS) among study participants, categorized by gender and overall percentages. The x-axis represents three IDDS categories: Lower (≤ 3), Medium (4 & 5), and Higher (≥ 6). The y-axis shows the percentage of participants in each category

The mean IDDS was 4.11 (*SD* = 1.29). Overall, most of the people living with HIV (56%) had ‘medium IDDS’ which is equivalent to “diet needing improvement’. About 31% (95%CI: 26.7% − 35.3%) of the participants actually had poor diet (‘lower IDDS’), with only about 14% having ‘higher IDDS’ (good diet). The prevalence of poor diet is higher among males than females; 40% (95%CI: 28.2% − 51.8%) and 29% (95%CI: 24.4% − 33.6%) respectively (Fig. 2).

### Determinants of diet quality

Males reported greater proportion of ‘low IDDS’ (poor diet) level (40%). Both males and females reported the same ‘higher IDDS’ (good diet) levels of 14%. The relationship between sex and IDDS was however not statistically significant (p = 0.172). There was also no association between IDDS and level of education, age of participants, and marital status. An association with a borderline significance was observed between marital status and IDDS (p = 0.049).

The only socio-demographic variable that showed a statistically significant association with IDDS was occupation. ‘Low IDDS’ (poor diet quality) was highest among farmers (50%), followed by PLHIV who were unemployed (44%), and then Artisans (35%). Formal sector workers (both public and private) reported the highest proportion of IDDS (good diet quality). The difference in IDDS (diet quality) between the different occupations was statistically significant (p < 0.05) (Table 3).

Other factors including alcohol consumption, exercise, whether they eat outside or not, meal frequency per day, ARV type, as well as duration of medication were also compared with IDDS. Only meal frequency was significantly associated with IIDS (*p* = 0.05).

**Table 3:** Determinants of diet quality (IDDS)

Factor	IDDS Category					Total, *n* (%)		*p*-value
	Low, *n* (%)		Medium, *n* (%)	Higher, *n* (%)				
**Age Category**								
18–34 years	16 (32.0)		28 (56.0)	6 (12.0)		50 (100.0)		0.106
35–54 years	63 (25.5)		147 (59.5)	37 (15.0)		247 (100.0)		
55 + years	54 (38.8)		68 (48.9)	17 (12.2)		139 (100.0)		
**Sex**								
Female	107 (28.8)		213 (57.4)	51 (13.7)		371 (100.0)		0.172
Male	26 (40.0)		30 (46.2)	9 (13.8)		65 (100.0)		
**Marital Status**								
Single	33 (25.8)		72 (56.3)	23 (18.0)		128 (100.0)		0.049
Married	29 (32.6)		55 (61.8)	5 (5.6)		89 (100.0)		
Divorced	13 (38.2)		17 (50.0)	4 (11.8)		34 (100.0)		
Widowed	40 (31.7)		72 (57.1)	14 (11.1)		126 (100.0)		
Separated	6 (46.2)		3 (23.1)	4 (30.8)		13 (100.0)		
Cohabiting	12 (26.7)		23 (51.1)	19 (22.2)		45 (100.0)		
**Level of Education**								
None	32 (29.4)		67 (61.5)	10 (9.2)		109 (100.0)		0.395
Primary	27 (32.1)		48 (57.1)	9 (10.7)		84 (100.0)		
Middle/JHS	61 (30.8)		102 (51.5)	35 (17.7)		198 (100.0)		
Secondary/SHS	9 (27.3)		18 (54.5)	6 (18.2)		33 (100.0)		
Higher	4 (33.3)		8 (66.7)	0 (0.0)		12 (100.0)		
**Occupation**								
Unemployed	21 (43.8)		21 (43.8)	6 (12.5)		48 (100.0)		**0.004**
Farmer	19 (50.0)		16 (42.1)	3 (7.9)		38 (100.0)		
Artisan	23 (35.4)		33 (50.8)	9 (13.8)		65 (100.0)		
Public Sector	2 (9.5)		12 (57.1)	7 (33.3)		21 (100.0)		
Private Sector	2 (22.2)		4 (44.4)	3 (33.3)		9 (100.0)		
Trading	66 (26.2)		155 (61.5)	31 (12.3)		252 (100.0)		
Others	0 (0.0)		2 (66.7)	1 (33.3)		3 (100.0)		
**Ever consumed Alcohol**								
YES	31 (31.3)	56 (56.6)			12 (12.1)		99 (100.0)	0.863
NO	102 (30.3)	187 (55.5)			48 (14.2)		337 (100.0)	
**Exercise**								
YES	78 (26.9)	172 (59.3)			40 (13.8)		290 (100.0)	0.058
NO	55 (37.7)	71 (48.6)			20 (13.7)		146 (100.0)	
**Eats outside?**								
No	20 (39.2)	23 (45.1)			8 (15.7)		51 (100.0)	0.251
Yes	113 (29.4)	220 (57.1)			52 (13.5)		385 (100.0)	
**Meal Frequency**								
At most twice	38 (35.2)	50 (46.3)			20 (18.2)		108 (100.0)	**0.05**
At least thrice	95 (29.1)	193 (59.0)			39 (11.9)		327 (100.0)	
**ARV type**								
First line	121 (31.4)	213 (55.3)			51 (13.2)		385 (100.0)	0.237
Second line	12 (23.5)	30 (58.8)			9 (17.6)		51 (100.0)	
**Duration of medication**								
< 6 months	10 (31.2)	20 (62.5)			2 (6.2)		32 (100.0)	0.225
6 months - <12 months	13 (48.1)	13 (48.1)			1 (3.7)		27 (100.0)	
1 – < 4 year	28 (29.8)	53 (56.4)			13 (13.8)		94(100.0)	
4 years and above	82 (29.0)	157 (55.5)			44 (15.5)		283 (100.0)	

This table presents a comprehensive analysis of various factors influencing the Individual Dietary Diversity Score (IDDS) across different demographic and behavioral groups among people living with HIV. It categorizes participants based on age, sex, marital status, level of education, occupation, lifestyle choices, and medication adherence, delineating their distribution across three diet quality categories: low, medium, and high IDDS. n = number of participants; *Significant at *p* < 0.01

### Associated factors of individual dietary diversity score (diet quality)

As presented in Table 4, we observe that an increase in the predictor variable age, was associated with lower odds of falling into a higher IDDS category (Adjusted Odds Ratio [AOR] = 0.966: 95%CI: 0.936–0.997: *p* = 0.031). suggests that, as individuals get older, they are less likely to maintain a diverse diet. This highlights the importance of targeted interventions for older PLHIV to promote dietary diversity.

Compared with cohabiting, PLHIV who are married (AOR = 4.634: 95%CI: 1.329–16.157: *p* = 0.0016) are more likely to belong to a higher IDDS category, whereas PLHIV who are widowed are less likely to belong to a higher IDDS category (AOR = 0.0203: 95%CI: 0.036–0.994: *p* = 0.049). Married PLHIV exhibited significantly higher odds of belonging to a higher IDDS category, potentially due to shared responsibilities and improved access to resources. Conversely, widowed individuals showed reduced odds, indicating potential challenges in maintaining dietary diversity after the loss of a spouse.

In terms of meal frequency per day, the probability of belonging to a higher IDDS category was 56% less for PLHIV who had two meals or less compared with those who had three meals or more per day (AOR = 0.441: 95%CI: 0.478–1.948: *p* = 0.020). The substantial decrease in the probability of belonging to a higher IDDS category for PLHIV with two meals or less emphasizes the critical role of regular and adequate meal frequency in achieving dietary diversity.

These findings collectively suggest that tailored interventions for different age groups, consideration of marital status in dietary support programs, and a focus on maintaining sufficient meal frequency could enhance nutritional outcomes and overall well-being among PLHIV.

No statistically significant associations were observed for sex, occupation, and exercise (*p* > 0.05).

**Table 4:** Associated factors of individual dietary diversity score (Diet Quality)

Characteristics	AOR	95% CI		*p*-value
**Age**	0.97	0.94–1.00		0.031
**Sex**				
Female	1.28	0.42–3.83		0.657
Male	*Ref*			
**Marital Status**				
Single	1.20	0.48–2.98		0.702
Married	4.63	1.33–16.16		0.016
Divorced	1.64	0.41–6.57		0.484
Widowed	1.99	0.73–5.46		0.181
Separated	0.20	0.04–0.99		0.049
Cohabiting	*Ref*			
**Occupation**				
Unemployed	1.26	0.08–19.05		0.869
Farmer	1.11	0.07–18.27		0.940
Artisan	1.08	0.08–14.66		0.956
Public sector	0.45	0.03–7.04		0.568
Private sector	0.65	0.03–14.33		0.788
Trading	1.40	0.10–19.04		0.799
Others	*Ref*			
**Do you exercise?**				
No	0.97	0.48–1.95		0.921
Yes	*Ref*			
**Meal frequency per day**				
At most twice	0.44	0.22 − 0.88		0.020
At least thrice	*Ref*			

This table presents the adjusted odds ratios (AOR) with their 95% confidence intervals (CI) and p-values for various demographic and lifestyle characteristics associated with the Individual Dietary Diversity Score (IDDS) among people living with HIV. *Ref. =* Reference group

### Dietary diversity (diet quality) and anthropometric/body composition parameters

As shown in Table 1, the results indicate a lack of significant association between dietary diversity and potential predictor variables such as body mass index (BMI), percentage body fat, and percentage muscle mass (*p* > 0.05). However, a noteworthy finding is the significant association observed between dietary diversity and factors like visceral fat and weight (*p* < 0.05).

Specifically, the results reveal an inverse relationship between visceral fat and dietary diversity, suggesting that a one-unit increase in visceral fat is associated with approximately 16.4% decrease in the odds of having a higher dietary diversity (diet quality). Conversely, a positive association was observed between weight and dietary diversity, where a one-unit increase in weight was associated with approximately 5.4% increase in the odds of having a higher dietary diversity (Table 1). These nuanced associations underscore the complexity of the relationship between dietary habits and various body composition parameters.

## Discussion

In this study, we evaluated the diet quality of people living with HIV (PLHIV) and assessed the demographic and other associated factors of diet quality. We found that diet quality among the PLHIV was influenced by age, marital status, and daily meal frequency.

From the results of this study, it is found that most of the respondents consumed starchy staples. This finding is comparable with the findings from one study that reported starchy staples as the most consumed food group [28]. Again, traditionally, African meals are predominantly roots and tubers, cereals and grains, and plantains, which are often complemented with soups, stews and sauces [28]. It is however important to note that the African diet has undergone some transitions, and it is now much lower in quality; highly processed, energy-dense, and low fiber and micronutrients [29].

Consuming starchy foods alone is not considered a balanced meal. Starchy foods are loved among the African population because they are reported to be the major cash crops among farmers in the region [28]. It can thus be appreciated why starchy staples were the most consumed food substances. Similar to the findings of this study, a Nigerian study reported that over a period of three weeks, carbohydrate-based foods were consumed almost every day of the week readily available and cheap, whereas fruits and vegetables were the least consumed [30].

There was poor consumption of fruits and vegetables among the PLHIV (< 20%). This is much lower compared with reported fruit and vegetables consumption of 52.6% among adult Ghanaians [31]. The phenomenon can be attributed to lack of access and/or unavailability of variety of foods (food insecurity) [32]. In one study, it was reported that although PLHIV had knowledge on the importance of nutrition, their knowledge did not translate into practice, as most respondents were noted to consume less quality diet [33], which may be due to lack of access, or other medical and social barriers.

It is noteworthy that, more males were identified to consume poor diet compared to their female counterparts. This is consistent with other studies conducted in Africa which also reported that females have better dietary diversity than males [34, 35]. A study conducted in Canada found that men consumed less fruits and vegetables compared to females [36]. Another study concluded that women consume more fruits and vegetables than males, who are noted for consuming pork, eggs and foods high in sucrose [37]. Further, women have been reported to seek nutritional counselling more often than men do [37], which may also explain the difference. Households that are headed by women enjoy higher dietary diversity (diet quality) than male-headed households [38], indicating that women are more conscious about their nutrition than males. It is worth noting however that, the current finding challenges often-held assumptions that women might face greater nutritional challenges due to societal or economic limitations affecting their resource access [39].

Understanding the nature of the diet of the study participants was necessary because type of food consumed is associated with development of certain chronic conditions [40]. Accordingly, a study reported that the type of diet consumed plays a role in an individual’s risk of developing Type 2 diabetes and other chronic conditions [41, 42]. Diabetes and cardiovascular diseases are reported to be responsible for about 17.9 million morbidities and mortalities, as well as some cancers [43].

Optimum nutrition plays a critical role in the care of PLHIV, especially in localities with limited nutritional resources [4]. It has been argued that in a continent like Africa, providing good quality diet is a challenge and malnutrition is a real problem in many developing countries [44]. This challenge may actually be worse with co-morbid conditions like HIV [4] and covid-19. Enhancing the nutritional status of PLHIV leads to improved HIV prognosis [33]. Thus, ensuring adequate diet is imperative for optimum health of PLHIV.

The findings of our study indicated that most PLHIV (56%) need to improve their diet (medium IDDS). This is similar to the findings of a study by Geofrey Maila et al., [45] in a similar setting in Zambia (rural) which reported that up to 64.4% of the respondent had diets needing improvement.

Our study further revealed that a significant proportion of the participants actually have poorer diet quality (31%), which is again comparable with the Zambian study (35.6%) [45]. The findings of our study may be related to an effect that antiretroviral medications are having on the appetite of the PLHIV resulting in the observed high consumption of energy dense foods and low fruits and vegetables intake, rendering their diet very low in quality. Conversely, one study among Chinese population reported a very high levels of IDDS, even though about eight different food varieties were identified among the PLHIV that participated in the study [4]. This could be explained by the difference in the socioeconomic income levels of the two countries; low-and middle income for Ghana, and upper middle income for China.

We observed in this study that about 14% of the respondents had high quality diet (higher IDDS) which is higher than the proportion reported in a Ugandan study that indicated the proportion of respondents with good quality diet to be 9% [46]. Food insecurity is now well acknowledged to increase HIV vulnerability as well as worsen PLHIV’s clinical outcomes [47, 48]. Access to sufficient, safe, and nutritious food that satisfies people’s dietary needs and food choices for an active and healthy life is a necessary condition for food security [49]. Food insecurity is linked to HIV-related mortality among PLHIV, partial HIV RNA suppression, long-term CD4 cell decline, increased opportunistic infections, and hospitalizations [48].

The World Health Organization recommends that people living with HIV (PLHIV) should strive to meet their micronutrient requirements by enhancing access to a diverse diet, fortified foods, and micronutrient supplements as needed [50]. Additionally, they suggest that in regions where micronutrient deficiencies are prevalent, initiatives should be undertaken to guarantee that people living with HIV (PLHIV) obtain all essential micronutrients through their dietary intake. Given the reported poor level of diet quality in this study, the call by the WHO is very much well placed for PLHIV in Ghana.

An ordinal logistic regression model was used to identify associated factors of diet quality (IDDS). The age of the PLHIV at the time of the interview predicted diet quality inversely. Every unit increase in the age of an HIV person was associated with 3.4% less likelihood of belonging to a higher diet quality category (AOR = 0.966, 95%CI: 0.936–0.997). This is expected because physiologically, aging is associated with changes to the intestinal tract and sensory function (depressed function of taste buds) which lead to poor appetite, reduced food intake, and inappropriate food choices [51, 52]. Our finding is however inconsistent with a study in South Africa, which reported that older people consume more veggies and grains [53]. This might be explained by the South African study being conducted during the rainy season, a period when vegetables are more likely to be in abundance.

Marital status has also been noted to influence dietary quality. Our findings show that PLHIV who are married have about 4.5 folds increased odds of having a good quality diet compared with PLHIV who are cohabiting (AOR = 4.634, 95%CI: 1.329–16.157). Similar observation was made in a study by Roos E et al., in which they concluded that compared to people who had previously been married, currently married people have dietary behaviour that aligns more with dietary recommendations [54]. Our study findings further reveal that PLHIV who are cohabiting also turn to have better quality diet as compared with PLHIV that separated (previously married). PLHIV who are separated have 0.2 less odds of having a good quality diet as against PLHIV who are separated (AOR = 0.203, 95%CI: 0.036–0.994). This discovery is unsurprising, as cohabiting and being married exhibit significant similarities, which may apply to their dietary behavior.

This study shows that diet quality of PLHIV was associated with daily meal frequency. PLHIV who had two or less meals per day were 55% less likely to have a good diet quality as compared with PLHIV who had at least three meals per day (AOR = 0.441, 95%CI: 0.221 − 0.879). This outcome is expected as having a higher frequency of meals per day could lead to consuming a more varied diet. This finding is corroborated by an Australian study that found that, for both men and women, the frequency of meals was positively correlated with micronutrient intakes and overall diet quality [men: OR = 5.6, 95%CI: 3.9–7.3); women: OR = 4.1, 95%CI: 2.2, 5.9)] [55]. The study therefore concluded that frequency of meals plays a significant role in determining nutrient intakes and the quality of diets.

Surprisingly, we did not find significant association between occupation and diet quality among the study participants, even though it was significant at the bivariate stage of the analysis. This means that other variables may have confounded occupation at the bivariate analysis stage. Our finding is however supported by a Japanese study that similarly found no significant association between one’s occupation and dietary intake [56]. Conversely, several studies have reported that occupation and higher socioeconomic status are associated with consumption of a more varied diet [57, 58].

The research also explored the connection between anthropometric and body composition parameters and dietary diversity. The findings reveal a positive correlation between dietary diversity and body mass index (BMI), indicating that a one-unit increase in BMI is associated with an approximately 13% likelihood of being in a higher category of dietary diversity. Although this association is not statistically significant (*p* > 0.05), the authors deem it clinically relevant. A similar observation is noted concerning the weight of the study participants, where a one-unit increase in participant weight corresponds to approximately a 5.4% chance of falling into a higher category of dietary diversity (*p* < 0.05). These outcomes align with the findings of several previous studies [22, 59, 60]. One potential explanation is that the consumption of a diverse range of foods might be linked to a higher intake of energy, given that many food groups contribute to overall energy consumption. This rationale again aligns with a perspective presented by Jayawardena et al., who observed that in Sri Lankan adults, an increase in dietary scores corresponded to an elevated percentage of consumption across most food groups. This pattern could potentially lead to an excess intake of energy and, consequently, contribute to obesity [61].

Participants’ visceral fat is another factor that has been explored in this study. Visceral fat exhibited a negative association with dietary diversity. In the present investigation, it was observed that a one-unit increase in visceral fat led to approximately a 16.4% reduction in the likelihood of belonging to a higher category of dietary diversity. This finding is supported by a study conducted in Ethiopia, which reported that adults who followed a less varied diet had a twofold higher likelihood of developing abdominal obesity compared to those who had a more diverse dietary pattern [AOR = 2.05, 95% CI: (1.31–3.19)] [62]. Moreover, in a cross-sectional study involving Iranian women aged 18 to 28 years, it was observed that a greater dietary diversity quartile was linked to reduced odds of both overall and abdominal obesity [63]. However, community-based cross-sectional investigations carried out among rural Asian Indians [64] and Sri Lankans [61] demonstrated a positive correlation between abdominal obesity and Dietary Diversity Score (DDS). In both studies, individuals with abdominal obesity exhibited higher DDS scores in comparison to non-abdominally obese groups. The variations in these findings may arise from distinct methodologies and population characteristics employed in assessing abdominal obesity, dietary intake, and determining DDS [23].

The relevance of this paper to the scientific community lies in the fact that it provides valuable information on the dietary habits of PLHIV in Ghana. This information can be used to develop targeted interventions to improve the diet quality of PLHIV in Ghana. The study also highlights the need for further research to identify other factors that may influence the dietary habits of PLHIV in Ghana.

The mean Individual Dietary Diversity Score (IDDS) of 4.11 indicates that, on average, the diet quality among PLHIV in Ghana is suboptimal. Moreover, the fact that a majority (56%) of the participants fall into the “diet needing improvement” category underscores the urgency of addressing dietary issues among this vulnerable population. This information is invaluable for healthcare practitioners who can use it to tailor nutritional counseling and interventions for PLHIV. Practitioners can benefit from this paper by using the findings to develop nutrition education programs for PLHIV in Ghana. The identification of associated factors of diet quality, such as age, marital status, and daily meal frequency, provides actionable insights. The findings suggest that age and marital status play significant roles in determining diet quality among PLHIV. This information can be used by healthcare practitioners to target specific age groups and marital status categories with tailored dietary interventions.

The study’s relevance extends to policy makers as well. With about 31% of the participants having a poor diet, there is a clear need for policy initiatives that focus on improving the nutritional status of PLHIV. Policy makers can use this data to develop evidence-based programmes and policies aimed at enhancing the dietary diversity and quality of PLHIV in Ghana, which can ultimately contribute to better health outcomes and quality of life for this population. The study can also help policy makers develop targeted interventions to improve the dietary habits of PLHIV in Ghana. One of the key findings from the study is that a significant proportion of PLHIV in Ghana primarily consume foods from the ‘Starchy staple’ group, while less than 20% of the participants reported consuming ‘Fruits’ and ‘Vegetables’ a day before the survey. This highlights the need for interventions aimed at diversifying the diets of PLHIV to ensure they receive a broader spectrum of essential nutrients.

The study had a few limitations. Firstly, due to unavailable contact information for many of the PLHIV, we used a sampling strategy which is well discussed in the methods section of this paper, that maintained randomness but excluded PLHIV not attending during data collection, potentially impacting the study’s generalizability. The sampling strategy may also limit the generalizability of the study findings, as it potentially excludes PLHIV who do not regularly attend clinic services, possibly skewing the sample towards those who are more health-conscious or in better health. This selection bias could make the results less reflective of the broader PLHIV population [65].

Again, the use of 24-hour recall also has drawbacks, including the inability of a single day’s intake to characterize an individual’s usual diet, and as is the case with all retrospective data collection, the main limitation generally cited for the 24-hour recall is its dependence on the ability of the subjects to adequately remember what they consumed and accurately report [66–68]. These could lead to under- or overestimation of intakes. Again, factors such as HIV respective information including date of HIV diagnosis or duration of HIV infection, viral load, CD4 count were not captured, which are factors that could potentially influence dietary diversity [69].

We estimated diet quality of the study participants using the Individual Dietary Diversity Score (IDDS) by the Food and Agricultural Organization (FAO). This does not capture quantities and specific nutrients consumed by the participants. Furthermore, we acknowledge that the cross-sectional design employed in our research confines us from establishing formal causal inferences. Cross-sectional studies capture a snapshot of data at a specific point in time, providing valuable insights into associations but precluding the determination of causality or the direction of relationships between variables. While our study has contributed valuable information on associated factors of diet quality among people living with HIV/AIDS in Ghana, we caution readers against interpreting the observed associations as indicative of causal relationships.

To enhance the representativeness and accuracy of future studies on the dietary habits of people living with HIV (PLHIV), several methodological improvements can be made. Extending the duration of data collection and employing community-based sampling methods could ensure a more diverse sample that includes PLHIV who are less engaged with healthcare facilities. This approach would help mitigate the potential selection bias of clinic-based sampling. Additionally, refining dietary assessment methods through the use of multiple 24-hour dietary recalls across various days, including weekdays and weekends, would offer a more accurate depiction of usual dietary intake. The adoption of technology-based tools for real-time food recording could further diminish recall bias, enhancing the reliability of dietary data collected.

Moreover, integrating comprehensive clinical data in research would enable more detailed analyses of how HIV-related health indicators such as viral load and CD4 count impact dietary practices and nutritional status. This would allow for a deeper exploration of the interactions between disease progression and diet quality. Expanding the measurement of diet quality to include detailed food quantity assessments and nutrient analyses would provide a fuller picture of nutritional adequacy and its impacts on health outcomes. Additionally, employing longitudinal study designs could address the limitations of cross-sectional approaches by tracking dietary changes and health outcomes over time, thereby facilitating a clearer understanding of causality and the dynamics of dietary practices among PLHIV.

Future research should undertake a comprehensive examination of the specific dietary patterns, nutritional deficiencies, and the impact of diet quality on the overall health and well-being of people living with HIV (PLHIV). It is essential to understand how different foods and nutrients affect the progression of the disease and the efficacy of treatment regimens over time. To this end, longitudinal studies would be invaluable as they could assess the long-term effects of dietary habits on disease progression and treatment outcomes among this population. Additionally, experimental studies designed to test the effectiveness of targeted nutritional interventions could provide critical insights. These interventions might include personalized dietary counseling, the provision of nutrient-rich foods that are known to support immune function, and comprehensive nutritional education programs specifically tailored to meet the unique needs of PLHIV in Ghana. Such research would not only fill gaps in current knowledge but also guide the development of policies and programs aimed at improving the nutritional status and overall health outcomes of PLHIV.

Healthcare practitioners working with people living with HIV (PLHIV) should prioritize routine dietary screening and assessment in their clinical practice. This approach is crucial for identifying individuals with suboptimal diet quality, enabling the development and implementation of personalized nutritional interventions and support tailored to the specific dietary needs and health circumstances of each patient.

Also, policy makers and other relevant stakeholders should develop and fund public health campaigns that focus on raising awareness about the importance of diet quality, especially among PLHIV. These campaigns should aim to change societal perceptions of nutrition and encourage healthier eating habits. Policies and programmes that improve access to affordable and nutrient-rich foods, including fruits and vegetables, for vulnerable populations, including PLHIV. This could involve subsidies, incentives for local agriculture, or targeted food assistance programmes. Deliberate efforts must be made for the collection of comprehensive data on diet quality among PLHIV to inform evidence-based policy decisions.

## Conclusion

This study demonstrates that most of the PLHIV did not consume a good diet which may have an implication on their immune system, as it is already under attack by the HIV itself, and probably emerging new infectious diseases such as COVID-19. Starchy staples were the most consumed food group among respondents, whereas fruits and vegetable consumption were considered unsatisfactory. Age, marital status, and daily meal frequency were the factors that predicted diet quality, accounting for about 20% of the variation in the diet quality of study participants. Overall, the study provides valuable insights into the dietary habits of PLHIV in Ghana. The findings can be used by the scientific community, practitioners, and policy makers to develop targeted interventions aimed at improving the diet quality of PLHIV in Ghana.

## Data Availability

The datasets used and/or analysed during the current study are available from the corresponding author on reasonable request.

## Data availability

Data is available upon request.

## Abbreviations

AIDS: Acquired Immunodeficiency Syndrome
ART: Antiretroviral Therapy
FAO: Food and Agriculture Organization
FQQ: Food Frequency Questionnaire
HIV: Human Immunodeficiency Virus
IDDS: Integrated Digital Delivery System
PLHIV: People Living with HIV
